# Muscle Metastasis as an Atypical Presentation of Non-small Cell Lung Cancer: A Case Report and Literature Review

**DOI:** 10.7759/cureus.86338

**Published:** 2025-06-19

**Authors:** Fatima Rezzoug, Jihane Derfoufi, Ouissam Al Jarroudi, Sami Aziz Brahmi, Said Afqir

**Affiliations:** 1 Medical Oncology, Mohammed VI University Hospital, Oujda, MAR; 2 Faculty of Medicine and Pharmacy, Mohammed First University, Oujda, MAR

**Keywords:** case report, gluteal mass, muscle metastasis, non-small cell lung cancer, skeletal muscle, soft tissue metastasis

## Abstract

Skeletal muscle metastases from non-small cell lung cancer (NSCLC) are rare and often clinically silent, representing an uncommon site of disease dissemination and accounting for approximately 2.3% of cases. Diagnosis is frequently delayed due to nonspecific symptoms and imaging findings that can mimic benign conditions.

We report the case of a 64-year-old man with a history of chronic smoking who presented with right gluteal pain and swelling. Imaging revealed a soft tissue mass within the gluteus maximus muscle. Histopathological analysis of a biopsy specimen demonstrated a poorly differentiated adenocarcinoma. Immunohistochemical staining was positive for CK7 and TTF-1 and negative for CK20, PSA, and CDX2, supporting a pulmonary origin. Molecular testing was negative for ALK, ROS1, RET, MET, and EGFR mutations. The patient received first-line chemotherapy with carboplatin and paclitaxel, achieving stable disease. He subsequently developed brain metastases, which were treated with whole-brain radiotherapy, and is currently undergoing second-line treatment with docetaxel.

This case highlights that gluteal muscle metastasis can present as the initial manifestation of NSCLC, preceding the detection of the primary tumor. It underscores the diagnostic value of imaging and immunohistochemistry in characterizing atypical soft tissue lesions. Multidisciplinary discussion is crucial for accurate diagnosis and optimal management of such rare presentations.

## Introduction

Lung cancer is the most common form of cancer and the primary cause of cancer-related deaths worldwide, with increasing rates of incidence, morbidity, and mortality [1,2]. The prognosis of lung cancer is often poor, largely due to delayed diagnosis and early metastasis [2]. In 50% of cases, non-small cell lung cancer (NSCLC) is detected at an advanced stage, often metastatic to the lung, liver, bone, adrenal glands, and central nervous system [3]. Metastasis to soft tissues, including skeletal muscle, is uncommon and not fully understood [4]. Autopsy series suggest that the overall incidence of skeletal muscle metastasis in cancer patients is approximately 0.08% [5]. The literature reports few cases of lung cancer metastasizing to soft tissues, with an overall prevalence estimated at 2.3% [6]. Soft tissue metastases (STMs) of lung cancer can manifest in a variety of sites, including the back muscles, chest, abdomen, thighs, and paraspinal muscles [4]. Nevertheless, STMs in carcinoma are rare due to the hostile environment in soft tissues that suppresses tumor cell proliferation [7]. These metastases typically develop late in the disease course but can sometimes appear before the primary tumor is detected, underscoring the importance of distinguishing them from other soft tissue masses [8]. To date, very few cases involving gluteal muscle metastasis from NSCLC have been reported in the literature, making this observation particularly uncommon. Here, we report a rare case of lung cancer metastasis to the gluteal muscle in a 64-year-old man.

## Case presentation

A 64-year-old man with a significant smoking history of 30 pack-years presented to our hospital with a progressively enlarging mass in the left gluteal region, which had been present for the past two months. On examination, a firm, spherical, non-pulsatile mass measuring approximately 1 cm was palpated deep within the left gluteal region. The mass was fixed, with irregular borders, and showed no signs of overlying skin changes or surrounding trauma. It was hard and painless to palpation.

**Figure 1:**
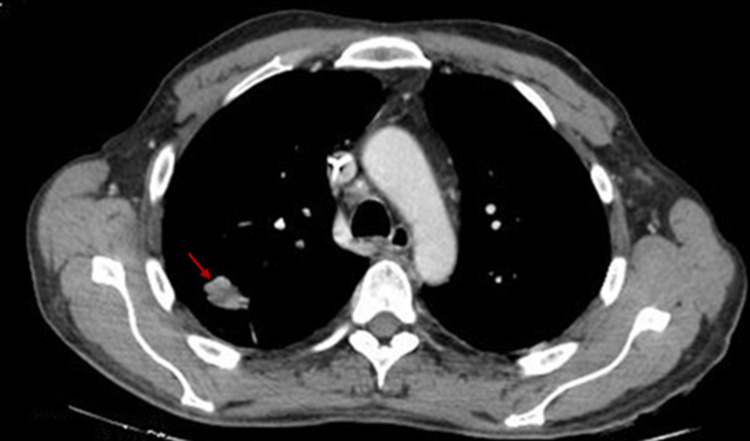
Axial contrast-enhanced chest CT scan. Axial CT image of the thorax showing a right upper lobe mass with soft tissue and intermediate density, located in the posterior segment. The lesion has irregular and microlobulated contours and measures approximately 22 × 13 mm.

**Figure 2:**
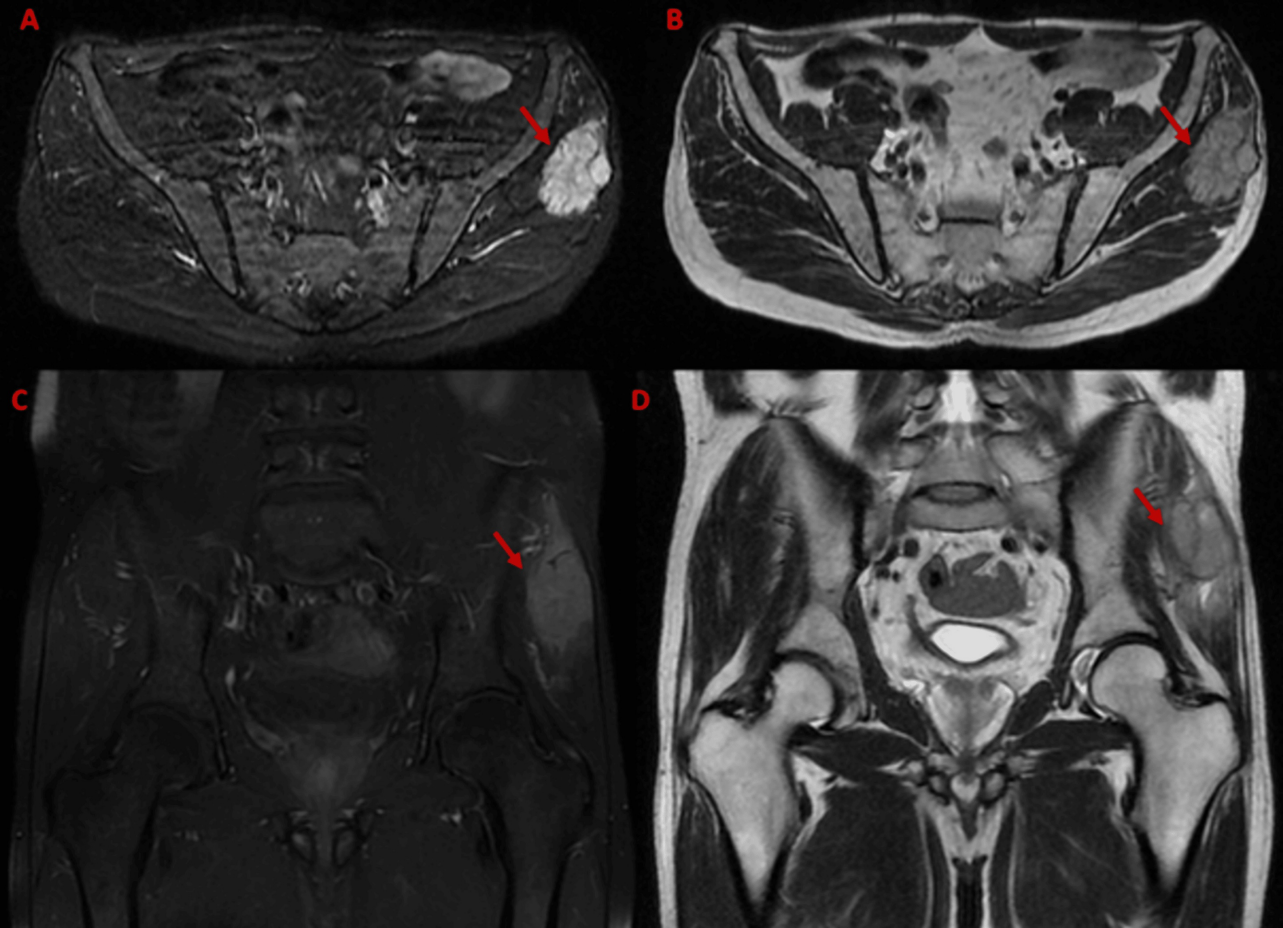
MRI evaluation of the right gluteal mass. (A) Axial T2-weighted fat-suppressed MRI sequence showing a hyperintense soft tissue lesion in the right gluteus maximus. (B) Axial T1-weighted image showing the lesion as isointense. (C) Coronal T2-weighted fat-suppressed sequence confirming the hyperintense signal of the lesion. (D) Coronal T1-weighted image revealing an isointense mass in the same location.

A full-body contrast-enhanced CT scan, including the brain, neck, chest, abdomen, and pelvis, revealed a soft tissue mass infiltrating the left gluteus medius muscle. Additionally, multiple bilateral pulmonary nodules were identified, some of which were calcified, along with mediastinal and hilar lymphadenopathy. A spiculated mass measuring approximately 22 × 13 mm was noted in the posterior segment of the right upper lobe, exhibiting soft tissue density and irregular, microlobulated contours (Figure 1). MRI confirmed a suspicious gluteal soft tissue mass (Figure 2). A PET-CT scan was not performed due to limited availability and financial constraints.

**Figure 3:**
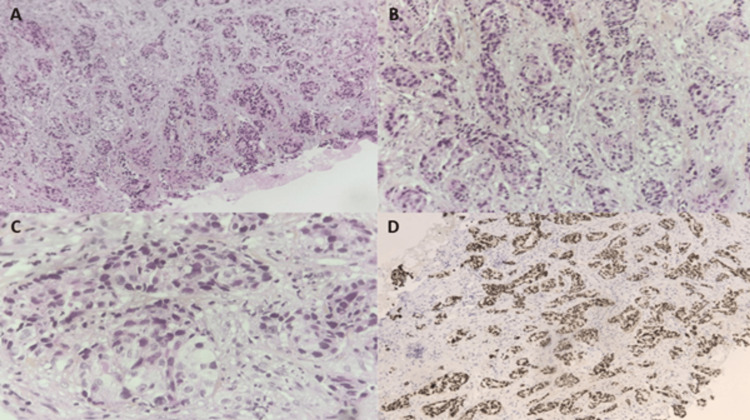
Histopathological and immunohistochemical examination of the gluteal mass. Histopathological evaluation demonstrating a poorly differentiated adenocarcinoma. Hematoxylin and eosin (H&E) staining, (A) ×100, (B) ×200, and (C) ×400. (D) Immunohistochemical staining showing positivity for TTF-1.

A core needle biopsy of the gluteal mass was performed under imaging guidance. Histopathological examination revealed a poorly differentiated adenocarcinoma, with tumor cells arranged in nests, cords, and occasional glandular structures. The cells exhibited moderate-to-marked cytonuclear atypia, hyperchromatic nucleolated nuclei, and abundant eosinophilic cytoplasm. Numerous mitotic figures were observed. Immunohistochemical staining demonstrated positivity for CK7 and TTF-1 and negativity for CK20, PSA, and CDX2, supporting a pulmonary origin (Figure 3). Molecular testing for ALK, ROS1, RET, MET, and EGFR mutations was negative for all examined exons. PD-L1 testing was not conducted, as the assay was not routinely available at our institution at the time of diagnosis. Additionally, next-generation sequencing (NGS) could not be performed due to limited access, representing a limitation in the molecular characterization of this tumor.

This case was discussed at our multidisciplinary tumor board, where the consensus was to initiate first-line chemotherapy with carboplatin (AUC 5) and paclitaxel (175 mg/m²) administered every three weeks. This regimen was selected based on the advanced stage of the disease, the absence of actionable driver mutations (ALK, ROS1, RET, MET, and EGFR all negative), and the patient's preserved performance status. The patient completed six cycles of chemotherapy, achieving stable disease, and was subsequently placed under clinical and radiological surveillance.

Six months later, the patient developed neurological symptoms, and brain imaging revealed multiple metastases. He underwent whole-brain radiotherapy and is currently receiving second-line systemic treatment with docetaxel.

## Discussion

Metastasis of lung cancer to soft tissues is considered uncommon. Here, we present the case of a 64-year-old man who initially presented with a mass in the left gluteal region. The biopsy confirmed a pulmonary origin.

The lung is the most prevalent location for cancer development, with more than half of cases diagnosed at an advanced stage [3]. Lung cancer can spread to any organ, typically through lymphatic or vascular routes. According to a recent study conducted by Salvatierra et al., individuals diagnosed with adenocarcinoma or large-cell carcinoma faced a greater likelihood of extrathoracic metastases compared to those with squamous cell carcinoma [9].

Metastasis of lung cancer tends to occur more frequently in the liver, brain, bone, and kidney, while STMs are relatively rare [3]. Lung cancer is the most prevalent primary malignant tumor that results in STMs [6]. These metastases can affect the skeletal muscle, subcutaneous tissue, and skin [8]. Skeletal muscle cancers are typically primary tumors rather than secondary tumors [10]. Despite muscles comprising nearly 50% of the body's weight, primary metastasis to the muscles is rare [3].

The precise occurrence of skeletal muscle metastasis remains uncertain, although findings from an autopsy series indicate it may be around 0.08% [5]. According to a study by Tuoheti et al., among a group of 2,557 patients diagnosed with lung cancer, skeletal muscle metastases were observed in only four individuals, accounting for a very small percentage (0.16%) [11]. While skeletal muscle metastasis is most frequently caused by lung malignancy, it may also occur from the gastrointestinal tract, prostate, bladder, kidney, pancreatic, thyroid, breast, or ovarian malignancies [12]. It is not entirely clear why metastatic tumors are so rare in skeletal muscle, but possible factors could include blood flow, metabolism, and high tissue pressure [13].

There have been various theories proposed to account for the infrequency of these lesions. Skeletal muscle poses challenges for tumor cells due to factors such as muscle contractions, lactic acid production, and fluctuating blood flow [13]. Additionally, skeletal muscle is capable of mounting a robust immune response, and its resistance to tumor implantation results in a gradual progression of metastasis [13].

The mechanism behind metastasis to distal soft tissues, such as skin and skeletal muscle, is not well understood; however, hematogenous spread is still regarded as the primary route for distant dissemination [14]. Other suggested mechanisms include lymphatic spread and trauma. Muscular dysfunctions due to trauma, for example, can reduce the muscle's ability to produce lactic acid, making it a more favorable environment for cancer cells [15]. Some reported cases in the literature have noted muscle metastases in areas previously affected by muscle trauma, which can delay diagnosis by mimicking common complications of the initial injury [15].

These metastases usually manifest in the context of a known neoplastic disease and are rarely the initial indicator of the primary tumor, as seen in our patient [8]. They are multifocal in 40% of cases and can theoretically affect any muscle [16]. In contrast, gluteal involvement is exceedingly rare. Our case stands out due to this unusual location, whereas most reported cases describe metastases to muscles such as the trapezius, adductor, psoas, or vastus [8,13,17].

Clinically, these metastases often present with variable pain, a tumor mass syndrome, and extra-articular stiffness [11]. In our patient, a firm, non-pulsatile 1 cm mass with irregular borders was palpated deep in the left gluteal region, without any skin changes or pain.

When muscle pain persists despite symptomatic treatment, it is important to consider other potential causes, including cancer, especially in individuals with risk factors for malignancies or those who have not kept up with regular cancer screenings. Diagnosing muscle metastases can be challenging due to their often small, microscopic size. MRI is the preferred imaging method for evaluating and detailing their extent [18]. Additionally, positron-emission tomography using 18F-fluorodeoxyglucose may help identify muscle metastases that were previously unnoticed [18]. A biopsy remains the definitive way to confirm the diagnosis [18].

Imaging can diagnose muscle tumors but cannot determine their metastatic nature. On ultrasound, muscle metastases appear as heterogeneous hypoechoic images with varying borders [18]. Computed tomography (CT) reveals a spontaneously hypodense tissue mass that enhances heterogeneously with contrast, with possible necrotic and calcified areas [18]. MRI shows these lesions as isodense in T1 sequences and hyperdense in T2 sequences, surrounded by peripheral edema, with a strong signal enhancement after contrast injection [18]. However, these features are non-specific and can be seen in primary tumors, abscesses, hematomas, or intramuscular collections.

Detection of soft tissue metastasis may have prognostic implications and provide more accessible biopsy sites. This can ultimately help avoid invasive procedures when internal neoplasms are suspected.

Histopathological diagnosis of muscle metastases can be made by needle biopsy under ultrasound or CT guidance or by surgical biopsy, allowing for en bloc tumor resection [18].

Soft tissue metastasis in lung cancer can have a significant impact on staging and prognosis, suggesting an advanced primary cancer. Patients with soft tissue metastasis secondary to lung cancer typically have a maximum median survival of four months [14]. According to a study by Di Giorgio et al., the presence of lung cancer metastasis to skeletal muscle significantly decreases the survival rate when compared to cases without metastasis [19]. While uncommon, the presence of muscular metastasis from primary lung carcinoma is often associated with a poor prognosis [8]. It is crucial to take into account the potential diagnosis of muscular metastases in patients who exhibit uncommon muscle pain or masses, even though it is a rare occurrence. Early detection of these metastases is vital, as it can guide the clinical approach and potentially improve patient management and outcomes.

STMs are primarily treated with chemotherapy and radiotherapy, with surgery reserved for select cases [20]. For muscle metastases, there is no standardized treatment regimen. However, surgical excision and/or local radiotherapy, sometimes supplemented with chemotherapy, can help prolong survival if the primary tumor is controlled [18]. Managing these lesions involves surgical removal for symptomatic relief and oncological control, followed by radiotherapy for pain relief and local control [17]. Overall, treatment is often palliative, aiming to improve quality of life by reducing muscle pain.

This variability in clinical presentation, anatomical location, and therapeutic approach is well documented in the literature. Table 1 summarizes selected reported cases of skeletal muscle metastases from lung cancer, highlighting the diversity in histological subtypes, muscles affected, clinical manifestations, and treatment modalities.

**Table 1:** Summary of reported cases of skeletal muscle metastases from lung cancer.

Author (year)	Histological type	Muscle involved	Presentation	Treatment
Strauss et al. (2012) [13]	Squamous cell carcinoma	Psoas muscle	Chest pain	Concurrent chemoradiotherapy and local radiotherapy in the muscle
Kwas et al. (2013) [17]	Small-cell carcinoma	Trapezius	Painless neck mass	Chemotherapy
Kwas et al. (2013) [17]	Squamous cell carcinoma	Adductor	Bone pain	Chemotherapy
Putro et al. (2024) [8]	Adenocarcinoma	Vastus muscles (thigh)	Painful thigh mass	Targeted therapy
This study (2025)	Adenocarcinoma	Gluteal muscle	Painful gluteal mass	Chemotherapy

## Conclusions

Muscle metastases from NSCLC are rare but clinically significant, often presenting asymptomatically and posing diagnostic and therapeutic challenges. Their detection, particularly through advanced imaging modalities, can significantly alter the treatment approach. This case underscores the aggressive nature of NSCLC with muscle involvement, highlighting the poor prognosis associated with such metastatic patterns. Histological examination remains essential for definitive diagnosis, especially in atypical presentations, and immunohistochemistry plays a crucial role in determining the primary origin. Additionally, multidisciplinary tumor board discussions are key to guiding personalized management decisions in such complex and uncommon clinical scenarios.
